# Rabies virus-based COVID-19 vaccine CORAVAX™ induces high levels of neutralizing antibodies against SARS-CoV-2

**DOI:** 10.1038/s41541-020-00248-6

**Published:** 2020-10-16

**Authors:** Drishya Kurup, Christoph Wirblich, Holly Ramage, Matthias J. Schnell

**Affiliations:** 1 Department of Microbiology and Immunology, Sidney Kimmel Medical College, Thomas Jefferson University, Philadelphia, PA 19107 USA; 2 Jefferson Vaccine Center, Sidney Kimmel Medical College, Thomas Jefferson University, Philadelphia, PA 19107 USA

**Keywords:** Preclinical research, Infectious diseases

## Abstract

The recently emerged coronavirus SARS-CoV-2, the causative agent of COVID-19, is rapidly spreading in the world. The exponentially expanding threat of SARS-CoV-2 to global health highlights the urgent need for a vaccine. Herein we show the rapid development of a novel, highly efficient, and safe COVID-19 vaccine using a rabies virus-based vector that has proven to be an efficient vaccine against several emerging infectious diseases. This study reports that both a live and an inactivated rabies virus containing the SARS-CoV-2 spike S1 protein induces potent virus-neutralizing antibodies at much higher levels than seen in the sera of convalescent patients. In summary, the results provided here warrant further development of this safe and established vaccine platform against COVID-19.

## Introduction

Severe acute respiratory syndrome coronavirus 2 (SARS-CoV-2), a zoonotic pathogen belonging to the *Betacoronavirus* family, emerged in December 2019 in Wuhan, China. SARS-CoV-2 rapidly engulfed the world in a coronavirus disease (COVID-19) pandemic infecting more than 22.4 million people and resulting in at least 789,455 deaths (Johns Hopkins University, accessed August 20, 2020)^1^. Among the seven coronaviruses that cause respiratory disease in humans, four cause only mild infection (229E, NL63, OC43, and HKU1), and three are highly pathogenic (SARS-CoV, MERS, and SARS-CoV-2). SARS-CoV-2 most likely originated in bats and was transmitted to humans via an intermediary animal host, as has been shown for the other highly pathogenic human Coronaviruses MERS and SARS-CoV^2^. The molecular determinants behind the high transmissibility and pathogenicity of SARS-CoV-2 are still hypothetical, but the acquisition of a furin cleavage site in the spike protein as well as mutations in the receptor binding domain which allow the spike protein to bind to human angiotensin-converting enzyme (ACE2) appear to be critical/important factors^3–5^. The presence of these and perhaps other molecular signatures have made SARS-CoV-2 the most easily transmissible of the three pathogenic Coronaviruses. Unlike SARS, SARS-CoV-2 will probably not be eliminated or even contained until an effective vaccine becomes available.

ACE2 receptors have been found to mediate cellular entry of SARS-CoV-2 as well as other coronaviruses, including NL63 and SARS-CoV, with which SARS-CoV-2 shares a 76% amino acid identity^5^. Cells expressing ACE2 are susceptible to the SARS-CoV-2 Spike (S) glycoproteins, which project from the surface of the SARS-CoV-2 virion membrane and act as ligands^2^. In humans, neutralizing antibodies and/or T-cell immune responses are raised against several SARS-CoV-2 proteins but mainly target the S protein, suggesting that S protein-specific immune responses play an important part in protection^6^. Therefore, most vaccine approaches currently use the SARS-CoV S protein, or part of it, as the vaccine immunogen^7^.

More than 139 different vaccines against COVID-19 are currently in preclinical development; of these, thirty are in clinical trials^8,9^. These vaccines can be allocated to one of three different platforms: synthetic vaccines based on DNA or RNA; virus-like particles; or inactivated virions or viral vectors expressing part of the SARS-CoV-2 genes^10^. While an effective vaccine candidate is yet to be identified, the most promising candidates can be expected to induce virus-neutralizing antibodies that have been described as a hallmark for protection against coronaviruses^11^.

Here we present a rabies virus (RABV)-based vaccine that offers a combination of features that could prove valuable for an effective, globally distributed SARS-CoV-2 vaccine. Additionally, we have demonstrated protection against another coronavirus, MERS-CoV, in challenge studies in mice utilizing the same RABV vector platform^12^. Our previous work has proven that both live and chemically inactivated RABV vaccines are safe for animals^13,14^. Furthermore, since the RABV vaccine often provides life-long immunity, long-term stability of the SARS-CoV-2 immune responses will be assessed in future studies.

## Results

**Fig. 1:**
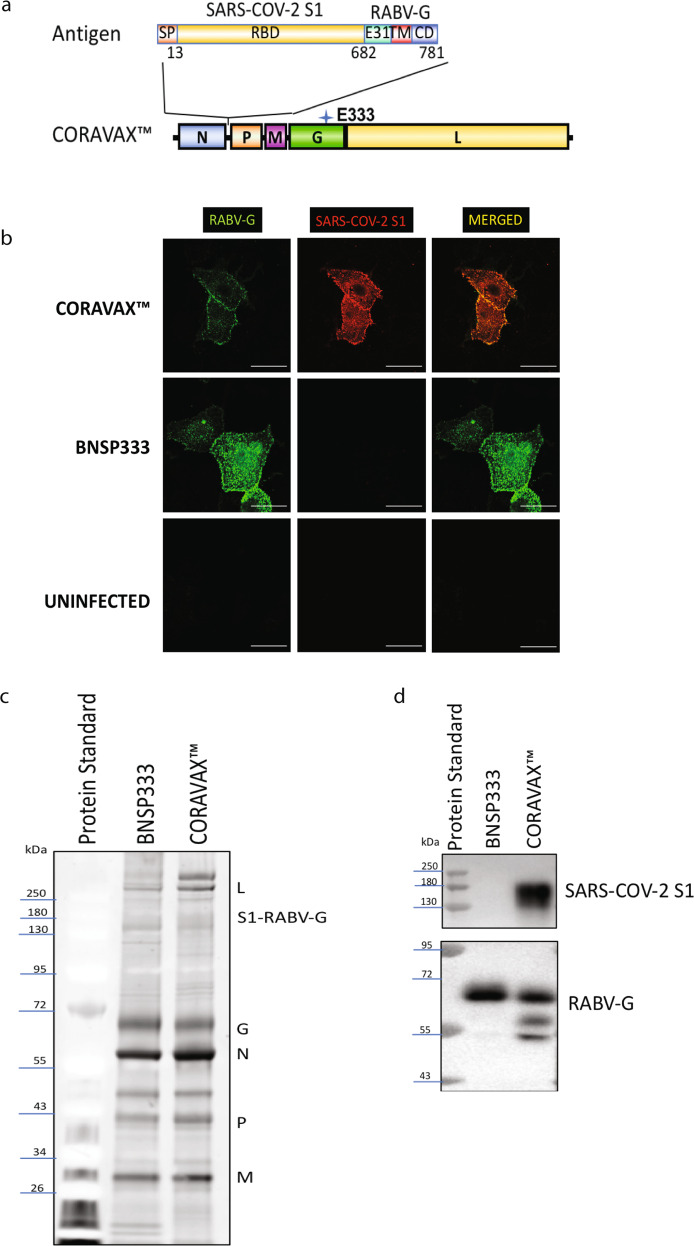
Characterization of the CORAVAX vaccine. **a** Schematic illustration of CORAVAX, the rabies virus-based SARS-CoV-2 vaccine construct used in this study. A SARS-CoV-2 S1 RABV G chimeric protein cDNA was inserted between the N and P genes of the SAD-B19-derived RABV virus vaccine vector BNSP333. **b** Immunofluorescence staining of Vero cells at 48 h post-infection labeled for either the RABV G protein (green) or the SARS-CoV-2 S1 protein (red).Scale bar represents 30 μm. **c** SDS-PAGE analysis of purified virions after sucrose gradient purification. Letters indicate the positions of the RABV L, G, N, P, and M and the chimeric S1-RABV-G proteins. **d** The panel shows western blotting of purified CORAVAX or control RABV(BNSP333) particles probed with anti-SARS-CoV-2 S rabbit polyclonal antibody or a human monoclonal 4C12 antibody directed against RABV G. All blots derive from the same experiment and were processed in parallel. Full size gels and WB are presented in the Supplemental material.

We introduced the SARS-CoV-2 S1 protein into the attenuated RABV vector based on our previous work with MERS-CoV (Fig. 1a). The RABV vaccine vector that we utilized derives from the attenuated RABV SAD-B19 vaccine strain^15^. Several modifications were introduced into the parent strain to increase its safety and maximize the expression of foreign genes. As previously shown, the expression of foreign antigens between the RABV N and P gene, as well as target gene codon optimization for human cells, results in the highest expression level of the foreign antigen^13^. Previous research with full-length MERS-CoV S also indicated that the full-length S protein of coronaviruses reduces viral titers dramatically, so we utilized the S1 subunit of the SARS-CoV-2 S. More importantly, the S1 domain containing the receptor-binding domain (RBD) is less conserved than the S2 fusion domain (64% vs. 90% identity with SARS-CoV)^16^.

To promote the incorporation of the S1 domain; we prepared a fusion protein between SARS-CoV-2 S1 and RABV G. Toward this approach the N-terminal 681 aa of SARS-CoV-2 S1 were fused to a truncated RABV glycoprotein, which comprises 31 aa of the ectodomain (ED) of RABV G and the complete cytoplasmic domain (CD) and transmembrane domain of RABV G to allow chimeric glycoprotein incorporation into RABV virions. The use of the transmembrane domain, as well as the cytoplasmic domain, has been shown previously to promote incorporation in RABV particles^12^. The chimeric SARS-CoV-2 S1/RABV G protein utilized the original SARS-CoV-2 ER translocation sequence (SS) and was generated by PCR of codon-optimized cDNA fragments. This construct was named CORAVAX™ (Fig. 1a). CORAVAX was recovered from cDNA on BSR or VERO cells, and viral stocks were prepared as described in the method section.

Next, we characterized the recombinant viruses. Vero cells were infected at a multiplicity of infection (MOI) of 0.01 with CORAVAX or empty RABV vector (BNSP333) or mock-infected. CORAVAX-infected cells stained for both RABV-G (green) and SARS-CoV-2 (red), while the empty RABV vector BSP333 stained only for RABV-G and the mock-infected cells did not stain (Fig. 1b). Importantly, both RABV G and SARS-CoV-2 S1 are expressed at similar regions of the cell surface, indicating the likelihood of co-incorporation into the virions, an essential requirement for the use of such recombinant particles as an inactivated vaccine. To further analyze the incorporation of the two glycoproteins, supernatants from CORAVAX- or vector BNSP333- infected VERO cells were concentrated and purified over a 20% sucrose and separated by SDS-PAGE. As shown in Fig. 1c, all five RABV proteins were detected for both CORAVAX and BNSP333. For CORAVAX, however, an additional protein band migrating at the expected size of 150 kDa for S1 was detected. Utilizing a SARS-CoV-2 specific antibody, we confirmed the presence of the chimeric SARS-CoV-2 -RABV G fusion protein as well as RABV-G by western blotting (Fig. 1d). In CORAVAX, we also observed differentially glycosylated RABV-G, which may be attributed to the addition of the S1 domain (Fig. 1d).

**Fig. 2:**
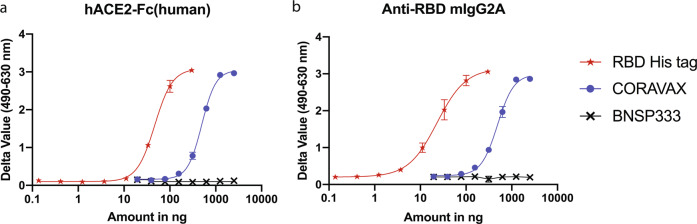
Conformational characterization of chimeric S1 in CORAVAX. Binding of a constant amount of **a** recombinant hACE2-Fc chimera and **b** Anti-RBD mIgG2a antibody to a dilution series of CORAVAX, RBD-His tag protein and BNSP333.

While the biochemical characterization of CORAVAX confirmed the successful incorporation of chimeric S1 into rabies virus particles, the antigen’s conformation was not addressed. To assess the correct confirmation of the chimeric S1 incorporated into CORAVAX, we first analyzed the binding of the recombinant human ACE-2 receptor-Fc chimera (human IgG) protein (Fig. 2a) and a SARS-COV-2 receptor binding domain (RBD) directed mouse monoclonal antibody (Fig. 2b). As shown in Fig. 2a, b, both the RBD antibody and the recombinant human ACE-2 receptor-Fc chimera protein bind to CORAVAX in a dose-dependent manner, indicating the proper folding of the S1 domain. The positive control RBD-His tag bound to the RBD mouse monoclonal and the hACE-2 receptor-Fc chimera protein while the negative control BNSP333 showed no signal.

**Fig. 3:**
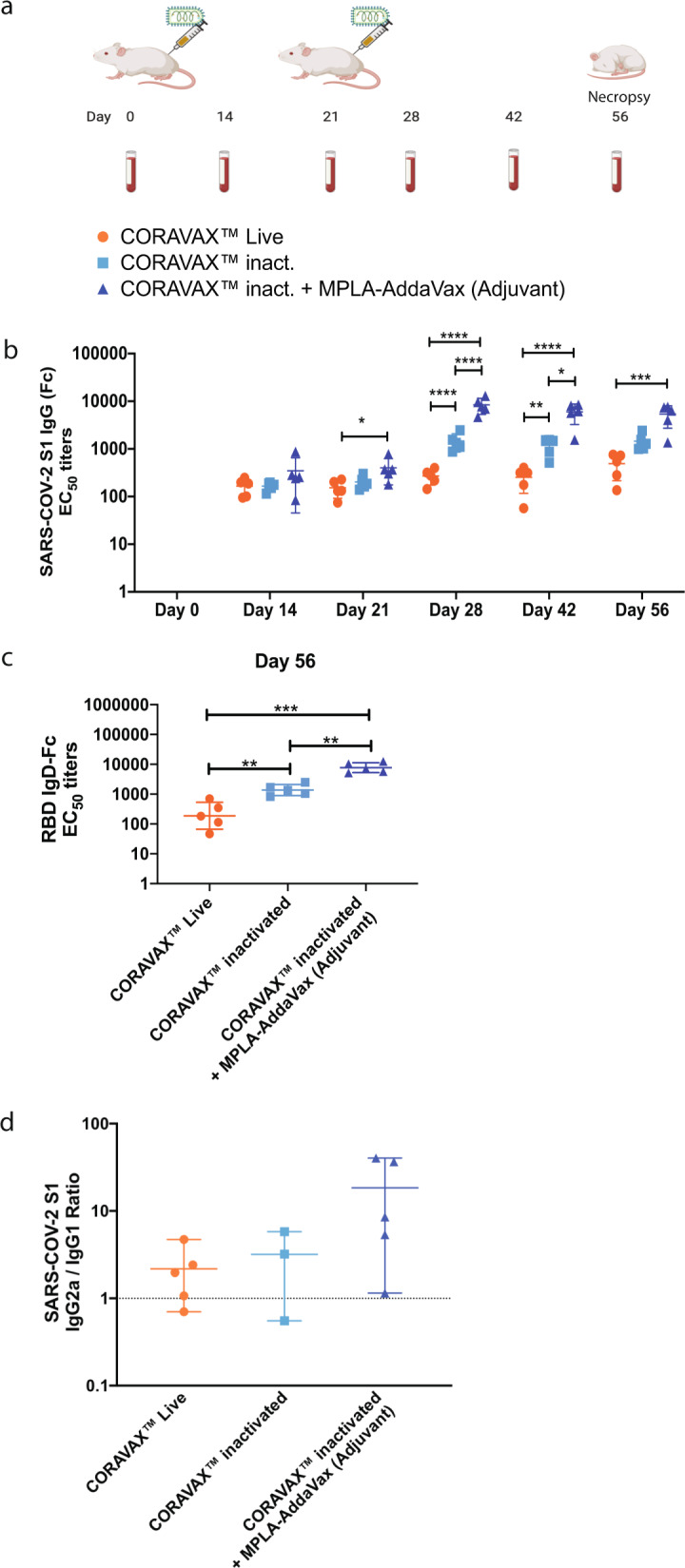
Analysis of the immune response to CORAVAX in mice. Balb/C mice were immunized once with live CORAVAX or twice (days 0 and 21) with 10 μg chemically inactivated particles of CORAVAX with or without adjuvant. Serum was collected from each mouse at days 14, 21, 28, 42, and 56 for analysis by a SARS-CoV-2 S-specific ELISA. **a** Immunization schedule: live CORAVAX used once on day 0; the inactivated vaccines were dosed on days 0 and 28 (figure created with BioRender.com). **b** SARS CoV-2 S1 IgG responses represented as EC50 titers **c** SARS CoV-2 RBD IgG responses on day 56 sera **d** SARS CoV-2 S1 IgG2/1 isotype ratio. Two animals in the inactivated CORAVAX group did not make detectable IgG1 antibodies.

To study the immunogenicity of the CORAVAX vaccine, we immunized groups of five BALB/c mice with live 10^7^ foci forming units (ffu) CORAVAX at day 0; 10 μg inactivated CORAVAX or 10 μg inactivated CORAVAX virions adjuvanted with MPLA-AddaVax at day 0 and 21 (Fig. 3a). Serum was collected on days 0,14, 21, 28, and 56 after the first immunization and analyzed for the presence of IgG against SARS-CoV-2 S by SARS-CoV S1-specific ELISA assay, as described in the methods section. All three vaccine approaches induced seroconversion and antibodies directed against the SARS-CoV-2 S (Fig. 3b). The detected humoral immune responses increased about ten-fold after the boost at day 21 for the inactivated vaccines. Interestingly, even the immune responses induced by the single inoculations with the live RABV slightly increased over time, indicating the medium-term immunostimulatory nature of the vector. To confirm the ELISA results observed with the recombinant SARS-CoV-2 S1, we performed ELISA’s utilizing the SARS-CoV-2 RBD as a target. Of note, antibodies detected against RBD have been indicated to predict virus neutralization antibodies (VNA) against SARS-CoV-2^17–20^. The results shown in Fig. 3c suggest that the immune responses were very similar to the S1 ELISA with the highest responses detected for animals immunized twice with adjuvanted CORAVAX and the lowest detected for the live viral vector.

Th1 responses are beneficial for protection against viral diseases and such responses have been shown to prevent pathology in the SARS-CoV S based vaccine model, we examined the induction of IgG isotypes after immunization at day 56. The live viral CORAVAX vaccine induced a balanced Th1/Th2 response while the killed vaccine with or without the adjuvant induced a more pronounced Th1-biased response (Fig. 3d).

Having shown that our vaccine CORAVAX is immunogenic, we further analyzed the potency of the induced antibodies. Development of virus neutralizing antibodies (VNA) have shown some correlation of protection against SARS COV-2^11,21,22^. As shown in Table 1, panel A, the sera (each group was pooled) from the live and inactivated vaccinated animals induce a high level of SARS-CoV-2 neutralizing antibodies, which aligns well with the SARS-CoV-2 S1 antibodies detected by ELISA, e.g., higher antibody titers detected in the S1-specific ELISA resulted in higher VNA. Both the adjuvanted and unadjuvanted vaccines resulted in high SARS-CoV-2 VNA, blocking 100% of the infection at a dilution of 1:600–1:1200, which is 20-fold more elevated than two different human convalescence sera we included as controls in the VNA assay. Sera collected from a patient during acute infection showed SARS-CoV-2 VNA was higher but still below the level in the sera from vaccinated mice. The presence of VNA is undoubtedly the first step to identify a potentially useful vaccine. However, reports that pathology can occur even in the presence of VNA will need to be addressed in an appropriate animal system^23,24^.

**Table 1:** Virus neutralizing antibodies against SARS-CoV-2 and RABV.

A		
Serum – mice pooled	Day 0 anti-SARS-CoV-2 VNA	Day 56 anti-SARS-CoV-2 VNA
CORAVAX live	0	1:250
CORAVAX inact.	0	1:600
CORAVAX inact. + MPLA-AddaVax	0	1:1200
BNSP333 (RABV Vector)	N/A	0
**Serum – human**	**anti-SARS-CoV-2 VNA**	
Convalescent serum human 1	1:35	
Convalescent serum human 2	1:27.5	
Acute infection human serum	1:240	
Pre COVID-19 RABV vaccinated human serum	0	

B		
Serum – mice pooled	Day 0 anti-RABV VNA	Day 56 anti-RABV VNA
CORAVAX live	0 IU	6.7 IU
CORAVAX inact.	0 IU	20 IU
CORAVAX inact. + MPLA-AddaVax	0 IU	140 IU
BNSP333 (RABV Vector)	N/A	46.7 IU
**Serum – human**	**anti-RABV VNA**	
Convalescent serum human 1	0 IU	
Convalescent serum human 2	0 IU	
Acute infection human serum	0 IU	
Pre COVID-19 RABV vaccinated human serum	50 IU	

Sera from CORAVAX vaccinated mice neutralize SARS-CoV-2 and RABV. Neutralization assays analyzed pooled sera from immunized mice against SARS-CoV-2 on Vero cells. The dilution at which SARS-CoV-2 infection was no longer detected by immunostaining against SARS-CoV-2 is shown as an average for two independent experiments (Table 1, panel A). Rabies neutralizing titers were assesed by RFFIT assay and represented as international units (IU) (Table 1, panel B).

We have previously demonstrated that the RABV-based Ebola virus (EBOV) vaccine immune responses observed in mice translate well to nonhuman primates (NHPs), at least against RABV G^13,25^. Because the expression of SARS-CoV-2 reduced expression of RABV G on the cell surface and also reduced the incorporation of RABV G into the RABV virus (Fig. 1d), we performed VNA assays against RABV. The results shown in Table 1, panel B indicate that all animals immunized with either the live, inactivated or inactivated and adjuvanted CORAVAX vaccines developed high VNA titers against RABV. Similar to the antibodies detected against SARS-CoV-2, the RABV VNA levels induced by single vaccination with the live CORAVAX vaccine were the lowest (about 6.7 IU), and the prime-boost immunization with the adjuvanted CORAVAX vaccine induced the highest level (averaging 140 IU). Of note, all three approaches resulted in RABV VNA levels way above the WHO protective titer of 0.5 IU.

## Discussion

The results presented in this preclinical study indicate that CORAVAX is an excellent candidate vaccine against COVID-19. While several SARS-CoV-2 vaccine candidates are at an advanced clinical phase, concerns for their efficiency remain. There have been concerns that the urgent need for a COVID-19 vaccine has pushed several vaccines into human trials without sufficient immunogenicity and a potential increase in antibody-mediated pathology studies. The most advanced mRNA vaccine has no animal data or clinical evaluation published, and information is dependent on company press releases. The DNA vaccine platform from two groups tested their vaccine responses in mice, guinea pigs, and NHPs and provoked immunogenicity but low VNA titers^26,27^. Another lead candidate, the adenovirus type 5 (Ad5) vector expressing SARS-CoV S, has shown to be immunogenic but induced low VNA titers in humans^28^. In another study, the SARS-CoV-2 challenged animals continued to have some residual viral RNA in the nasal swabs of ChAdOx1 nCoV-19 vaccinated NHPs, raising the concern of vaccinated humans still being infectious^29^. Another factor to consider is the longevity of the induced immune responses. Adenovirus based vector previously analyzed as an EBOV vaccine failed to induce long-term immunity, and the protective responses against EBOV wore off three months after immunization, requiring a boost with an unrelated vaccine vector^30^.

The utilization of the S1 domain, rather than the full-length S, might avoid concerns of binding of S2-specific non-neutralizing antibodies enhancing inflammation and potentially causing severe pathology. Certain antibody–virus immune complexes could bind to activating Fc receptors on alveolar macrophages, inducing the expression of pro-inflammatory factors, including IL-6 and CCL2. The antibody–virus immune complexes could also activate the complement system, which could further exacerbate inflammation^1^. Previous research suggests that more than 90% of humans already have antibodies against the four human coronaviruses (HCoVs, e.g., 229E, OC43, NL63, HKU1)^31^. The several highly conserved regions between the S2 domain of the four human coronaviruses with that of SARS-CoV-2 are potential targets for cross-reactive antibodies^32^. Therefore, immunization with the SARS-CoV-2 S protein might enhance these conserved epitopes and prevent a response against new epitopes in the S1 domain. Although not observed for coronaviruses, it has been demonstrated that pre-existing immunity in flaviviruses-experienced people can significantly shape their B-cell response against future flavivirus infections^33^.

Besides the immunogenicity of a vaccine, there are two concerns for the use of COVID-19 vaccines in humans: antibody-dependent enhancement (ADE), as seen previously for RSV^34^, and the vaccine-associated enhanced respiratory disease (VAERD). While there is little evidence of ADE for coronaviruses, probably because macrophages are not the primary target of human coronaviruses, there are concerns of VAERD. Vaccine-induced S-specific immunity induced after vaccination with a modified vaccinia virus Ankara (MVA) resulted in severe acute lung injury (ALI) in SARS-CoV challenged Chinese macaques compared to vector MVA controls^24^. Such adverse effects need to be addressed in animal systems before the mass vaccination of humans should be pursued.

The success rate of the development of vaccines based on viral vectored vaccines have been low, with the exception of the live VSV based EBOV vaccine^35^. The significant advantage of CORAVAX is that it is an inactivated vaccine based on the rabies vector that has been safely and effectively administered to pregnant women, children, elderly, and the immunocompromised along with inducing durable responses (CDC, MMWR). Also, the inactivated rabies vectored EBOV vaccine, FiloRab1 has provided 100% protection against EBOV challenge in NHP studies^25^. In summary, we have presented a novel vaccine against COVID-19 that is highly immunogenic in an animal model. The detected VNA titers induced by CORAVAX were much higher than the VNA detected in human convalescent sera^36^. While this first proof of concept study utilized a high dose of live virus (10^7^ ffu), studies with lower doses of live virus and different amount of the killed vaccine will be analyzed later. Of note, recent studies with adenoviruses or DNA vaccines used viral titers up to 10^11^ pfu and amount of up to 100 micrograms RNA.

Further characterization of the immune response by different vaccine doses and schedules to achieve protection, analyze potential toxicity are currently performed, and the vaccine is now manufactured. The first clinical studies are expected to begin in October 2020.

## Methods

### Cells and viruses

BSR, Vero E6 cells, and Vero (CCL-81) were obtained from ATCC and were cultured in 1X DMEM (Corning, Cat# 10-013-CV), supplemented with 5% (v/v) fetal bovine serum (FBS), 1% (v/v) penicillin/streptomycin, and were maintained at 37 °C and 5% CO_2_.

SARS-CoV-2 was obtained from BEI (WA-1 strain). Stocks were prepared by infection of Vero E6 cells in 2% serum for five days, freeze-thawed, and clarified by centrifugation (PO). The titer of stock was determined by plaque assay using Vero E6 cells and were 1 × 10^7^ pfu/mL and 1.5 × 10^6^ TCID50/mL. This seed stock was amplified in Vero CCL81 (P1) at 1.5 × 10^6^ TCID50/mL. All work with infectious virus was performed in a Biosafety Level 3 laboratory and approved by the Institutional Biosafety Committee and Environmental Health and Safety.

### Antibodies

SARS-COV/COV-2 S1 rabbit polyclonal sera obtained from Invitrogen, Cat# PA5-81798. SARS-CoV-2 S1 mouse polyclonal sera were obtained from mice immunized with a VSV vector expressing SARS-CoV2 S1. 4C12 Anti-RABV-G human monoclonal was produced from 4C12 hybridoma (provided by Dr. Scott Dessain, Lankenau Institute for Medical Research, Wynnewood, PA).

### Plasmid construction

The vaccine vector BNSP333 has been described previously^37^. Codon-optimized cDNA encoding SARS-CoV-2 Spike protein and RABV glycoprotein were obtained from Genscript. PCR fragments encoding amino acids 1-682 of SARS-CoV-2 and amino acids 428–524 of the SAD-B19 RABV glycoprotein were amplified using primers CP1674P 5′-AAACTAACACCCCTCCGTACGCCACCATGTTCGTGTTTCTGG-3′ and CP1675M 5′-CTCCCACAGACCTTGGGGAGTTTGTCTGGG-3′ and primers CP1676P 5′-CTCCCCAAGGTCTGTGGGAGATGAGGCCG-3′ and CP1677M 5′-TTAGTTTTTTTCATGGCTAGCTCACAGCCTGGTCTCGCC-3′, respectively. The two fragments were joined and inserted between the BsiWI and NheI sites of BNSP333 using In-Fusion cloning (Takara Bio Inc., Mountain View, CA) to create BNSP333-S1-RVG (CORAVAX). Recombinant clones were verified by DNA sequencing.

Codons 16–681 of the spike protein gene were amplified using codon-optimized cDNA as template and primers CP1699P: 5′-TGACGCACCTAGATCTGTGAACCTGACCACAAGGACC-3′ and CP1700M: 5′-CGTATGGATAGTCGACCCTTGGGGAGTTTGTCTGGG-3′ and inserted between the BglII and SalI sites of the pDISPLAY vector to generate the expression plasmid for production of soluble S1 fused to a C-terminal HA tag (Invitrogen Inc., Waltham, MA).

### Virus purification, inactivation and titration

Recombinant RABV were recovered, purified, inactivated and titered. Briefly, X-tremeGENE 9 transfection reagent (Millipore Sigma, Cat# 6365809001) was used to cotransfect the full-length viral cDNA clone encoding CORAVAX along with the plasmids encoding RABV N, P, and L proteins and the T7 RNA polymerase into BEAS-2B human lung cells in 6-well plates (RABV). Eight days post transfection supernatant was collected, filtered through a 0.22 µm membrane filter (Millipore) and titrated on VERO cells. The presence of recombinant virus was verified by immunostaining with monoclonal antibody against the nucleoprotein (FujiRebio, Cat# 800-092) and polyclonal antiserum against the S1 domain (Thermo Fisher, Cat# PA581798). The filtered virus was then used to inoculate VERO cells seeded in Cellstack Culture Chambers (Corning) and propagated in VP-SFM medium (Thermo Fisher Scientific) over a period of 18 days. Supernatant collected on day 10 post infection was filtered through 0.45 µm PES membrane filters (Nalgene) and layered onto 20% sucrose in DPBS. Virions were sedimented by ultracentrifugation in a SW32 rotor for 1.5 h at 25,000 rpm. Viral particles were resuspended in phosphate-buffered saline (PBS) and inactivated with 50 μl per mg of particles of a 1:100 dilution of β-propiolactone (BPL, Millipore Sigma, Cat# P5648) in cold water. The absence of infectious particles was verified by inoculating BSR cells with 10 μg of BPL-inactivated viruses over 3 passages.

For titration, a rabbit polyclonal antibody directed against the S1 subunit of SARS Coronavirus (ThermoFisher, Cat# PA581798) was used for staining overnight. The next day the cells were washed with DPBS and incubated with a mixture of AlexaFluor 555 conjugated anti-rabbit-IgG (ThermoFisher, Cat# A32794) and Dylight 488 conjugated monoclonal antibody 4C12 (Dr. Scott Dessain) directed against the RABV glycoprotein. After overnight staining, the cells were washed with DPBS, and fluorescent foci counted to determine viral titers.

### Characterization of sucrose purified virions

Purified virus particles were denatured in 4× Laemmli Buffer (BIO-RAD, Cat# 161-0747) supplemented with 10% beta-mercaptoethanol (w/v) at 95 °C for 5 min. Then 2.5 μg of total protein were resolved on a 10% SDS-polyacrylamide gel and thereafter stained overnight with Sypro Ruby for total protein analysis or transferred onto a nitrocellulose membrane in Towbin buffer (192 mM glycine, 25 mm Tris, 20% methanol) for western blot analysis. The nitrocellulose membrane was then blocked in PBST (1X PBS, 0.05% Tween-20) containing 5% (w/v) non-fat dried milk at room temperature for 1 h. After blocking, the membrane was incubated overnight with a human monoclonal 4C12 specific for the RABV glycoprotein (hybridoma kindly provided by Dr. Scott Dessain) or rabbit serum against the S1 subunit of SARS-CoV-2 Spike protein (Invitrogen, ThermoFisher Scientific, Cat# PA5-81798) at a dilution of 1:1000 in PBS containing 5% BSA. After washing, the western blot was incubated for 1 h with anti-human IgG HRP (for RABV-G) at 1:20,000 or anti-rabbit-IgG HRP (for SARS-CoV-2 S1) diluted 1:10,000 in blocking buffer. Bands were developed with SuperSignal West Dura Chemiluminescent substrate (Pierce, ThermoFisher Scientific Cat# 34075). Images were captured using a FLuorChem R CCD imaging system (ProteinSimple). Color Prestained Protein Standard, Broad Range (NEB #P7719) was used for molecular weight determination. All gels or blots were derived from the same experiment and were processed in parallel.

### Immunofluorescence

Vero E6 cells were seeded on coverslips in 1× DMEM (5% FBS) and infected the following day with CORAVAX™ or control RABV (BNSP333) in 1X DMEM (2% FBS) at 34 °C for 48 h. At the end of the incubation, the cells were washed with 1X PBS, fixed in 2% PFA for 30 min, and blocked with PBS containing 5% FBS. The cells were then stained for 2 h at RT with mouse polyclonal antiserum against the S1 subunit of SARS-CoV-2 Spike protein and a human monoclonal antibody 4C12 against RABV glycoprotein (2 µg/ml). Following washing with PBS and incubation with Cy3-conjugated anti-rabbit IgG HRP (1:200 in PBS containing 5% FBS, Jackson ImmunoResearch, West Grove, PA, Cat# 711-035-152) and Cy2 conjugated anti-human IgG HRP (1:250 in PBS containing 5% FBS, Jackson ImmunoResearch, Cat# 109-225-088) secondary antibodies. The cells were mounted in ProLong™ Glass Antifade Mountant with NucBlue™ Stain (Invitrogen, Cat# P36983). Images were taken using Nikon A1R+ confocal microscope. Composite images were prepared using Fuji.

### Conformational characterization of chimeric S1 in CORAVAX

Purified inactivated CORAVAX or control vaccine were coated in 3-fold decreasing concentrations starting at 25 μg/mL. Control antigen RBD-His was coated in 3-fold decreasing concentrations, starting at 300 ng/well A constant amount of SARS COV/2 Anti-receptor binding domain (RBD) mouse IgG2a antibody (InvivoGen, Cat # srbd-mab10, 1 μg/mL) or the recombinant human ACE-2 Fc chimera (human IgG, Biolegend, Cat # 793208, 1 μg/mL) were added to the wells in 1% Milk in 1× PBST (0.05%Tween-20). Secondary antibodies, goat anti-mouse IgG-Fc HRP (Southern Biotech, Cat# 1033-05, 1:2000 in PBST) or goat Anti human IgG-Fc HRP (Jackson ImmunoResearch, Cat# 109-035-098, 1:2000 in PBST) were used to detect the bound RBD mouse IgG2a antibody and human ACE-2 Fc chimera (human IgG) protein respectively.

Optical density was measured at 490 nm and 630 nm using an ELX800 plate reader (Biotek Instruments, Inc., Winooski, VT). Background subtracted data (OD430 - 630 nm) were analyzed with GraphPad Prism (Version 6.0 g) using 4-parameter nonlinear regression.

### Animal ethics statement

This study was carried out in strict adherence to recommendations described in the Guide for the Care and Use of Laboratory Animals, the Office of Animal Welfare, and the United States Department of Agriculture. All animal work was approved by the Institutional Animal Care and Use Committee (IACUC) at Thomas Jefferson University. All procedures were carried out under isoflurane anesthesia by trained personnel and under the supervision of veterinary staff. Mice were housed in cages in groups of five, under controlled humidity, temperature, and light (12 h light/12 h dark cycles) conditions. Food and water were available ad libidum.

### Immunizations

Six- to -eight-week-old female BALB/c mice were purchased from Charles River Laboratories (Wilmington, MA). Two groups of five mice were inoculated intramuscularly with 10 µg of chemically inactivated CORAVAX™ particles, with and without adjuvant (5 µg MPLAs in 2.5% AddaVax, Invitrogen, vac-mpls and vac-adx-10, respectively) in 100 µl PBS and boosted once with the same amount of virus ± adjuvant on day 21, respectively. Another group of five mice was immunized once with 10^8^ ffu of live CORAVAX™. Blood samples were collected by retro-orbital bleed before the first immunization until day 56 post-immunization.

### Recombinant proteins for ELISA

#### Purification of the HA-tagged protein from the supernatant of transfected cells for ELISA

Sub-confluent T175 flasks of 293T cells (human embryonic kidney cell line) were transfected with a pDisplay vector encoding amino acids 16 to 682 of SARS-CoV-2 S (S1) fused to a C-terminal hemagglutinin (HA) peptide using X-tremeGENE 9 reagent (Millipore Sigma, Cat# 6365809001). Supernatant was collected 6 days post-transfection, filtered through 0.22 um PES membrane filters (Nalgene) and the loaded onto an anti-HA agarose (Pierce, Cat# 26182) column equilibrated in PBS. After washing with ten bed volumes of PBS the column was loaded with 2 column volumes of HA peptide at a concentration of 400 µg/ml in PBS and incubated overnight at 4 °C. The following day protein was eluted with 2 column volumes of HA peptide followed by two column volumes of PBS. Fractions were collected and analyzed by western blotting with polyclonal antiserum against the S1 domain (Thermo Fisher, Cat# PA581798). Peak fractions were then pooled and dialyzed against PBS in 10,000 molecular weight cutoff (MWCO) dialysis cassettes (Thermo Fisher Scientific) to remove excess HA peptide. After dialysis, the protein was quantitated by UV spectrophotometry and frozen in small aliquots at −80 °C.

#### Purification of the RBD-His protein for ELISA

RBD-HIS^38^: The SARS COV-2 RBD-His tagged plasmid was purchased from Bei Resources (NR-52309). Sub-confluent T175 flasks of 293T cells (human embryonic kidney cell line) were transfected with the RBD-His tagged plasmid using X-tremeGENE 9 reagent (Millipore Sigma, Cat# 6365809001). Supernatant was collected 6 days post-transfection, filtered through 0.22 μm PES membrane filters (Nalgene). The 5 mL HisTALON cartridge (Clontech Laboratories, Cat # 635683) column was equilibrated with ten column volumes of Equilibration Buffer (HisTALON™ Buffer Set, Clontech Laboratories, Cat# 635651). The filtered supernatant was loaded onto the HisTALON cartridge (Clontech Laboratories, Cat# 635683) column at a speed of 1 mL/min. After washing with ten column volumes of Wash Buffer (Wash Buffer prepared by mixing 6.6 parts of Elution Buffer with 93.4 parts of Equilibration Buffer of the HisTALON™ Buffer Set), the sample is eluted (at a flow rate of ~1 ml/min) with approximately eight column volumes of Elution Buffer, collecting 1 ml fractions. The sample protein concentration was assessed by measuring the absorbance of the eluted fractions at 280 nm (Nanodrop, Thermo Fisher Scientific). Eluted fractions were analyzed by Western blotting with a mouse monoclonal RBD specific antibody (InvivoGen, Cat# srbd-mab10). Peak fractions were then pooled and dialyzed against PBS in 10,000 molecular weight cutoff (MWCO) dialysis cassettes (Thermo Fisher Scientific). After dialysis, the protein was quantitated by UV spectrophotometry and frozen in small aliquots at −80 °C.

#### ELISA

To determine antibody responses to the S protein of SARS-CoV-2, an indirect ELISA was developed utilizing purified S1 or receptor binding domain (RBD) protein. The production of the recombinant proteins is described above. Humoral responses to SARS-CoV-2 S1 and RBD protein were measured by an indirect ELISA. We tested individual mouse sera by enzyme-linked immunosorbent assay (ELISA) for the presence of IgG specific to SARS-Cov-2 S1 or RBD. In order to test for anti-SARS COV-2 S1 humoral responses, we produced soluble S1 or RBD as described above. The two recombinant proteins were resuspended in coating buffer (50 mM Na_2_CO_3_ [pH 9.6]) at a concentration of 0.5 μg/mL of S1 or 2 μg/mL of RBD and then plated in 96-well ELISA MaxiSorp plates (Nunc) at 100 μl in each well. After overnight incubation at 4 °C, plates were washed three times with 1× PBST (0.05% Tween 20 in 1× PBS), which was followed by the addition of 250 μl blocking buffer (5% dry milk powder in 1× PBST) and incubation at room temperature for 1.5 h. The plates were then washed three times with PBST and incubated overnight at 4 °C with serial dilutions of sera (in triplicate) in 1× PBST containing 0.5% BSA. Plates were washed three times the next day, followed by the addition of goat anti-mouse-IgG-Fc HRP antibody (Southern Biotech, Cat# 1033-05, 1:8000 in PBST) or goat anti-mouse-IgG1-Fc HRP antibody (Jackson ImmunoResearch, Cat# 115-035-205, 1:8000 in PBST) or goat anti-mouse-IgG2a-Fc HRP antibody (Jackson ImmunoResearch, Cat# 115-035-206, 1:8000 in PBST) for 2 h at RT. After the incubation, plates were washed three times with PBST, and 200 μl of *o*-phenylenediamine dihydrochloride (OPD) substrate (Sigma) was added to each well. The reaction was stopped by the addition of 50 μl of 3 M H2SO4 per well. Optical density was determined at 490 nm (OD_490_) and 630 nm (OD_630_) using an ELX800 plate reader (Biotek Instruments, Inc., Winooski, VT). Plates were incubated for 15 mins (IgG-Fc) or 10 mins (IgG2a or IgG1) with OPD substrate before the reaction was stopped with 3 M H2SO4. Data were analyzed with GraphPad Prism (Version 8.0 g) using 4-parameter nonlinear regression to determine the titer at which the curves reach 50% of the top plateau value (50% effective concentration [EC50]).

### SARS-COV-2 neutralization assays

Neutralizing virus titers were measured in serum samples that had been heat-inactivated at 56 °C for 30 min. Duplicates of each serum dilution were prepared in 96-well round-bottom plates starting at 1:50 dilution for mouse sera and 1:5 dilution for human sera in a total volume of 40 µl Optimem (1% Penicillin-Streptomycin). Vero CCL81 cells were plated in 96-well plates (100 µL/well) at a density of 25,000 cells per well. The following day, 100 pfu of SARS-CoV-2 was diluted into 30 µl DMEM and added to each dilution of serum samples. The serum and virus were incubated together at room temperature for 1 h and transferred to the supernatant of the Vero CCL81 cells. Cells were incubated under standard cell culture conditions at 37 °C and 5% CO_2_ for 48 h. Cells were fixed in 4% formaldehyde/PBS for 15 min at room temperature and then washed three times with PBST. Cells were blocked (2% BSA/PBST) for 60 min and incubated in primary antibody (anti-dsRNA J2) overnight at 4 °C. Cells were washed 3× PBS and incubated in secondary (Goat anti-mouse Alexa 488-ThermoFisher, Cat# A32723, and hoescht 33342-ThermoFisher, Cat# H3570) for 2 h at room temperature. Cells were washed 3× in PBST and imaged using ImagXpress Micro using a 10× objective. Four sites per well were captured, and wells were scored for viral infection. The assay was repeated twice (*N* = 2).

### RABV neutralization by rapid fluorescent-focus inhibition test

RABV virus neutralization was performed on BSR cells using CVS-11 strain with triplicates of each sample by standard methods. In brief: Serum was heat inactivated at 56 °C for 30 min. BSR cells (25,000 cells per well) were preseeded in a 96 well flat bottom plate 24 h prior to the assay. Serum samples were threefold serially diluted in triplicates in a 96-well round bottom plate, starting at a dilution of 1:30 for the mouse serum and 1:5 for the human serum. The U.S. standard rabies immune globulin was used at a starting dilution of 2 IU/mL. Working dilution of RABV CVS-11 strain (to get 90% infection) was prepared in 1X Optimem and 10 μL of the working dilution was added to each well containing the diluted serum. Plates were incubated for 1 h at 34 °C. Medium was aspirated from the 96-well plate preseeded with BSR cells and the diluted serum+ virus mix was transferred to the cells. Plates were incubated for 24 h at 34 °C. After the 24 h incubation, plates were fixed with 80% Acetone and stained with FITC-conjugated anti-RABV N antibody. 50% endpoint titers were calculated using the Reed -Muench method and converted to international unit (IU) per milliliter by comparing to the U.S. standard Rabies immune globulin.

### Statistical analysis

For the ELISA, log transformed 50% effective concentration (EC50) titers were plotting against delta OD value (OD 490–630 nm). One-way ANOVA with post-hoc Tukey HSD test was performed on log transformed data for each time point.

### Reporting summary

Further information on research design is available in the Nature Research Reporting Summary linked to this article.

## Supplementary information

Supplementary Information

Reporting Summary

## Data Availability

All data needed to evaluate the conclusions in the paper are present in the paper.
