# Supplementary figures and images for: Rabies virus-based COVID-19 vaccine CORAVAX™ induces high levels of neutralizing antibodies against SARS-CoV-2
Source: NPJ Vaccines. 2020 Oct 16;5:98. doi: 10.1038/s41541-020-00248-6 (PMC7568577; doi:10.1038/s41541-020-00248-6)

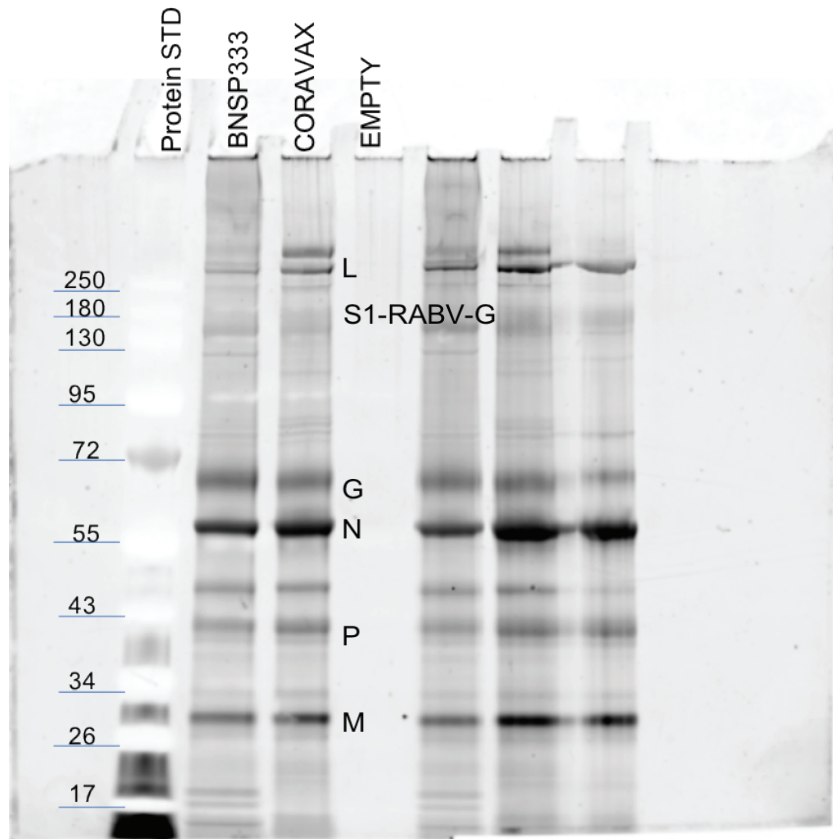

Figure 1c-Syrpro Ruby stained protein gel

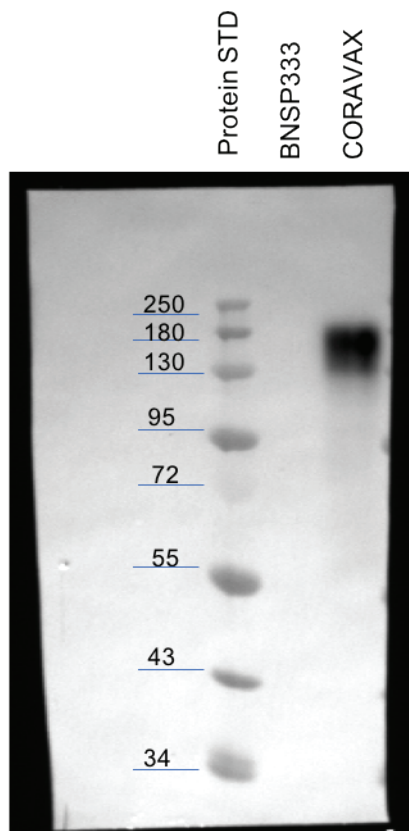

Figure 1d- WB probed for S1

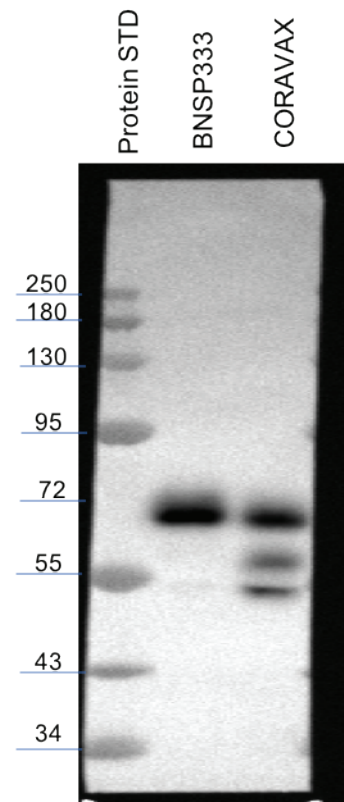

Figure 1d- WB probed for RABV-G

Supplement: Supplementary file 1 — Supplementary Information [file 41541_2020_248_MOESM1_ESM.pdf]
