# Supplementary material for: Rabies virus-based COVID-19 vaccine CORAVAX™ induces high levels of neutralizing antibodies against SARS-CoV-2
Source: NPJ Vaccines. 2020 Oct 16;5:98. doi: 10.1038/s41541-020-00248-6 (PMC7568577; doi:10.1038/s41541-020-00248-6)
Supplement: Supplementary file 2 — Reporting Summary [file 41541_2020_248_MOESM2_ESM.pdf]

nature research

Corresponding author(s): NPJVACCINES-00818R

Last updated by author(s): Aug 25, 2020

Reporting Summary

Nature Research wishes to improve the reproducibility of the work that we publish. This form provides structure for consistency and transparency in reporting. For further information on Nature Research policies, see our Editorial Policies and the Editorial Policy Checklist.

Statistics

For all statistical analyses, confirm that the following items are present in the figure legend, table legend, main text, or Methods section.

n/a	Confirmed	
☐	☒	The exact sample size (n) for each experimental group/condition, given as a discrete number and unit of measurement
☐	☒	A statement on whether measurements were taken from distinct samples or whether the same sample was measured repeatedly
☐	☒	The statistical test(s) used AND whether they are one- or two-sided<br>*Only common tests should be described solely by name; describe more complex techniques in the Methods section.*
☒	☐	A description of all covariates tested
☐	☒	A description of any assumptions or corrections, such as tests of normality and adjustment for multiple comparisons
☐	☒	A full description of the statistical parameters including central tendency (e.g. means) or other basic estimates (e.g. regression coefficient) AND variation (e.g. standard deviation) or associated estimates of uncertainty (e.g. confidence intervals)
☒	☐	For null hypothesis testing, the test statistic (e.g. F, t, r) with confidence intervals, effect sizes, degrees of freedom and P value noted<br>*Give P values as exact values whenever suitable.*
☒	☐	For Bayesian analysis, information on the choice of priors and Markov chain Monte Carlo settings
☒	☐	For hierarchical and complex designs, identification of the appropriate level for tests and full reporting of outcomes
☒	☐	Estimates of effect sizes (e.g. Cohen's d, Pearson's r), indicating how they were calculated

Our web collection on statistics for biologists contains articles on many of the points above.

Software and code

Policy information about availability of computer code

Data collection	N/A
Data analysis	N/A

For manuscripts utilizing custom algorithms or software that are central to the research but not yet described in published literature, software must be made available to editors and reviewers. We strongly encourage code deposition in a community repository (e.g. GitHub). See the Nature Research guidelines for submitting code & software for further information.

Data

Policy information about availability of data

All manuscripts must include a data availability statement. This statement should provide the following information, where applicable:

- Accession codes, unique identifiers, or web links for publicly available datasets
- A list of figures that have associated raw data
- A description of any restrictions on data availability

All data needed to evaluate the conclusions in the paper are present in the paper

Field-specific reporting

Please select the one below that is the best fit for your research. If you are not sure, read the appropriate sections before making your selection.

☒ Life sciences ☐ Behavioural & social sciences ☐ Ecological, evolutionary & environmental sciences

For a reference copy of the document with all sections, see nature.com/documents/nr-reporting-summary-flat.pdf

Life sciences study design

All studies must disclose on these points even when the disclosure is negative.

Sample size	Groups of five mice were determined as sufficient to see differences (or not) in the immune response after vaccination with the diffrent vaccines compared to each other as well as controls groups.
Data exclusions	No data were excluded
Replication	Groups of five mice were used and samples were analyzed in triplicate.
Randomization	The mice were randomized assigned to the cages by the laboratory animals services of TJU
Blinding	The VNA was performed blinded. The other immunogenicity assays were not performed blinded because the same researcher performed the immunization and sample collections and the ELISA assays.

Reporting for specific materials, systems and methods

We require information from authors about some types of materials, experimental systems and methods used in many studies. Here, indicate whether each material, system or method listed is relevant to your study. If you are not sure if a list item applies to your research, read the appropriate section before selecting a response.

Materials & experimental systems

n/a	Involved in the study	
☐	☒	Antibodies
☐	☒	Eukaryotic cell lines
☒	☐	Palaeontology and archaeology
☐	☒	Animals and other organisms
☒	☐	Human research participants
☒	☐	Clinical data
☒	☐	Dual use research of concern

Methods

n/a	Involved in the study	
☒	☐	ChIP-seq
☒	☐	Flow cytometry
☒	☐	MRI-based neuroimaging

Antibodies

Antibodies used	SARS-COV/COV-2 S1 rabbit polyclonal sera obtained from Invitrogen, Cat# PA5-81798, 4C12 Anti-RABV-G human monoclonal was produced from 4C12 hybridoma (provided by Dr. Scott Dessain, Lankenau Institute for Medical Research, Wynnewood, PA), AlexaFluor 555 conjugated anti-rabbit-IgG (ThermoFisher, Cat# A32794),Goat anti-human IgG HRP (Invitrogen, Cat # A18829),Donkey anti-rabbit-IgG HRP (Jackson ImmunoResearch, Cat# 711-035-152), Cy3-conjugated anti-rabbit IgG HRP (Jackson ImmunoResearch, , Cat# 711-035-152),Cy2 conjugated anti-human IgG HRP (Jackson ImmunoResearch, Cat# 109-225-088), SARS COV/2 Anti-receptor binding domain (RBD) mouse IgG2a antibody (InvivoGen, Cat # srbd-mab10, 1ug/mL), goat anti-mouse IgG-Fc HRP (Southern Biotech, Cat# 1033-05), goat Anti human IgG-Fc HRP (Jackson ImmunoResearch, Cat# 109-035-098),FITC-Anti RABIES N (Fujirebio, Cat no 800-092)
Validation	The antibodies are validated by the use of the appropriated controls such as uninfected cells or control vaccines, not containing the insert to be analyzed.

Eukaryotic cell lines

Policy information about cell lines

Cell line source(s)	VERO CCL81, ATCC; VERO-E6, ATCC; BSR cells, a BHK clone from the federal research center for viral diseases of animals, Germany.
Authentication	Has not been performed

Mycoplasma contamination	All cell lines did test negative for mycoplasma contamination in previous testing. Because contamination can occur each time the cells are used, this contamination can not be wholly excluded but is unlikely.
Commonly misidentified lines (See ICLAC register)	N/A

Animals and other organisms

Policy information about studies involving animals; ARRIVE guidelines recommended for reporting animal research

Laboratory animals	BALB/c mice, female
Wild animals	N/A
Field-collected samples	N/A
Ethics oversight	IACUC, Thomas Jefferson University

Note that full information on the approval of the study protocol must also be provided in the manuscript.
